# Supplementary material for: Distinct roles of amylin and oxytocin signaling in intrafamilial social behaviors at the medial preoptic area of common marmosets
Source: Commun Biol. 2023 Dec 5;6:1231. doi: 10.1038/s42003-023-05593-5 (PMC10698028; doi:10.1038/s42003-023-05593-5)
Supplement: Supplementary file 2 — Supplementary Information [file 42003_2023_5593_MOESM2_ESM.pdf]

Supplementary materials

Distinct roles of amylin and oxytocin signaling in within-family social behaviors at the medial preoptic area of common marmosets

Authors: Takuma Kurachi[1,2#], Kazutaka Shinozuka[1#], Chihiro Yoshihara[1,3], Saori Yano-Nashimoto[1,4], Ayako Y. Murayama[1,5, $], Junichi Hata[5,6,7], Yawara Haga[6], Hideyuki Okano[5,6], and Kumi O. Kuroda[1,3,8*]

[1] Laboratory for Affiliative Social Behavior, RIKEN Center for Brain Science, Saitama, Japan

[2] Department of Agriculture, Tokyo University of Agriculture and Technology, Tokyo, Japan

[3] School of Life Science and Technology, Tokyo Institute of Technology, Kanagawa, Japan

[4] Laboratory of Physiology, Department of Basic Veterinary Sciences, Graduate School of Veterinary Medicine, Hokkaido University, Hokkaido, Japan

[5] Department of Physiology, Keio University School of Medicine, Tokyo, Japan

[6] Laboratory for Marmoset Neural Architecture, RIKEN Center for Brain Science, Saitama, Japan

[7] Graduate School of Human Health Sciences, Tokyo Metropolitan University, Arakawa, Tokyo, Japan

[8] Laboratory for Circuit and Behavioral Physiology, RIKEN Center for Brain Science, Saitama, Japan

[#]: These authors contributed equally to this work.

$: Current address: Neural Circuit Unit, Okinawa Institute Science and Technology Graduate University, Okinawa, Japan

* **Contact**: Kumi O. Kuroda, School of Life Science and Technology, Tokyo Institute of Technology, 4259 Nagatsuta-cho, Midori-ku, Yokohama, Kanagawa 226-8503, Japan

Tel: +81-45-924-5652

e-mail: kurodalab@bio.titech.ac.jp

Supplementary files

Supplementary Table 1 Results of statistical analysis in infant retrieving assay with muscimol infusion into the cMPOA (related to Fig. 2)

	Main effects						Interaction effect		
	muscimol			cannula			muscimol:cannula		
	Chisq	Df	p value	Chisq	Df	p value	Chisq	Df	p value
Retrieval latency	61.720	1	3.96E-15 ***	1.569	1	0.2103	4.139	1	0.0419 *
Rejection rate	11.503	1	0.0007 ***	2.290	1	0.1302	0.259	1	0.6107
Carrying rate	103.960	1	2.06E-24 ***	1.160	1	0.2815	2.886	1	0.0894 †

Supplementary Table 2 Results of statistical analysis in family observation assay with muscimol infusion into the cMPOA (related to Fig. 3)

	Main effects						Interaction effect		
	muscimol			cannula			muscimol:cannula		
	Chisq	Df	p value	Chisq	Df	p value	Chisq	Df	p value
Carrying	0.5606	1	0.4540	3.4653	1	0.0627 †	0.6065	1	0.4361
Retrieval	1.2256	1	0.2683	1.6116	1	0.2043	0.4860	1	0.4857
Rejection	1.6374	1	0.2007	1.8560	1	0.1731	0.6447	1	0.4220
Body contact	1.0041	1	0.3163	0.7540	1	0.3852	2.2930	1	0.1300
Social play	1.6002	1	0.2059	0.4989	1	0.4800	0.6354	1	0.4254
Grooming	0.1987	1	0.6558	2.0381	1	0.1534	0.0797	1	0.7777
Being groomed	0.9656	1	0.3258	2.6783	1	0.1017	0.3802	1	0.5375
Vocalization	1.3777	1	0.2405	2.8539	1	0.0912 †	0.5479	1	0.4592
Locomotion	0.1092	1	0.7411	0.4680	1	0.4939	0.8063	1	0.3692
Eating	1.5014	1	0.2205	1.3563	1	0.2442	0.5947	1	0.4406
Drink	0.6241	1	0.4295	1.5190	1	0.2178	0.2481	1	0.6184
Sleep	0.7720	1	0.3796	0.3237	1	0.5694	0.3040	1	0.5814
Yawn	2.5531	1	0.1101	1.0159	1	0.3135	1.0074	1	0.3155
Self scratch	0.8923	1	0.3449	5.4721	1	0.0193 *	0.3568	1	0.5503
Self grooming	2.4881	1	0.1147	2.8864	1	0.0893 †	0.9849	1	0.3210
Stereotypy	0.7720	1	0.3796	0.3237	1	0.5694	0.3040	1	0.5814

Supplementary Table 3 Results of statistical analysis without Rika in family observation assay with muscimol infusion into the cMPOA (related to Fig. 3)

	Main effects						Interaction effect		
	muscimol			cannula			muscimol:cannula		
	Chisq	Df	p value	Chisq	Df	p value	Chisq	Df	p value
Carrying	4.2974	1	0.0382 *	1.5650	1	0.2109	0.4265	1	0.5137
Retrieval	2.1000	1	0.1473	1.2666	1	0.2604	0.1171	1	0.7322
Rejection	0.0211	1	0.8845	0.0963	1	0.7564	3.8836	1	0.0488 *
Body contact	2.8002	1	0.0943 †	0.2302	1	0.6314	0.7347	1	0.3914
Socia; play	1.6286	1	0.2019	0.0290	1	0.8648	0.1696	1	0.6804
Grooming	0.8747	1	0.3497	1.9296	1	0.1648	0.0885	1	0.7661
Being groomed	1.8051	1	0.1791	1.6008	1	0.2058	0.0118	1	0.9133
Vocalization	2.0988	1	0.1474	0.3598	1	0.5486	0.5662	1	0.4518
Locomotion	1.2745	1	0.2589	0.0579	1	0.8098	0.0303	1	0.8618
Eating	0.1460	1	0.7024	0.0024	1	0.9613	3.7201	1	0.0538 †
Drink	3.6972	1	0.0545 †	2.3460	1	0.1256	1.6622	1	0.1973
Sleep	1.1277	1	0.2883	3.0146	1	0.0825 †	2.8036	1	0.0941 †
Yawn	3.5410	1	0.0599 †	0.2003	1	0.6545	0.1678	1	0.6821
Self scratch	1.1766	1	0.2780	1.0654	1	0.3020	0.2345	1	0.6282
Self grooming	3.9258	1	0.0476 *	1.8790	1	0.1704	0.2574	1	0.6119
Stereotypy	0.8494	1	0.3567	0.5960	1	0.4401	3.3494	1	0.0672 †

Supplementary Table 4 Results of statistical analysis including sex in infant retrieving assay with muscimol infusion into the cMPOA (related to Fig. 2)

	Main effects								
	muscimol			cannula			sex		
	Chisq	Df	p value	Chisq	Df	p value	Chisq	Df	p value
Retrieval latency	61.920	1	3.58E-15 ***	1.338	1	0.2474	1.658	1	0.1979
Rejection rate	11.833	1	5.82.E-04 ***	2.673	1	0.1021	0.439	1	0.5076
Carrying rate	106.037	1	7.24E-25 ***	1.172	1	0.2789	0.259	1	0.6108

	Interaction effects											
	muscimol:cannula			muscimol:sex			cannula:sex			muscimol:cannula:sex		
	Chisq	Df	p value	Chisq	Df	p value	Chisq	Df	p value	Chisq	Df	p value
Retrieval latency	3.135	1	0.0766 †	2.728	1	0.0986 †	1.192	1	0.2750	0.116	1	0.7337
Rejection rate	0.015	1	0.9011	0.972	1	0.3242	1.722	1	0.1895	NA	0	NA
Carrying rate	2.269	1	0.1320	1.800	1	0.1797	0.199	1	0.6559	1.591	1	0.2071

Supplementary Table 5 Results of statistical analysis in family observation assay including sex with muscimol infusion into the cMPOA (related to Fig. 3)

	Main effects								
	muscimol			cannula			sex		
	Chisq	Df	p value	Chisq	Df	p value	Chisq	Df	p value
Carrying	1.460	1	0.2269	2.319	1	0.1278	0.057	1	0.8113
Retrieval	1.449	1	0.2287	0.866	1	0.3520	4.306	1	0.0380 *
Rejection	0.584	1	0.4448	0.828	1	0.3630	0.040	1	0.8418
Body contact	1.026	1	0.3112	0.006	1	0.9394	1.260	1	0.2617
Social play	2.790	1	0.0948 †	0.595	1	0.4406	0.064	1	0.8006
Grooming	1.027	1	0.3110	1.364	1	0.2428	0.012	1	0.9141
Being groomed	1.981	1	0.1592	1.433	1	0.2312	0.299	1	0.5847
Vocalization	0.866	1	0.3521	0.028	1	0.8671	0.656	1	0.4181
Locomotion	0.164	1	0.6855	0.019	1	0.8917	0.246	1	0.6199
Eating	3.722	1	0.0537 †	1.406	1	0.2358	0.179	1	0.6722
Drink	2.040	1	0.1532	0.195	1	0.6589	0.199	1	0.6557
Sleep	1.554	1	0.2125	4.213	1	0.0401 *	2.548	1	0.1105
Yawn	3.427	1	0.0641 †	0.315	1	0.5746	0.181	1	0.6703
Self scratch	0.898	1	0.3433	1.994	1	0.1579	1.772	1	0.1832
Self grooming	3.919	1	0.0478 *	2.559	1	0.1096	0.861	1	0.3535
Stereotypy	0.929	1	0.3351	0.606	1	0.4362	1.079	1	0.2990

	Interaction effects											
	muscimol:cannula			muscimol:sex			cannula:sex			muscimol:cannula:sex		
	Chisq	Df	p value	Chisq	Df	p value	Chisq	Df	p value	Chisq	Df	p value
Carrying	0.002	1	0.9613	0.435	1	0.5094	0.479	1	0.4891	0.177	1	0.6740
Retrieval	0.173	1	0.6778	1.828	1	0.1764	1.551	1	0.2130	0.002	1	0.9661
Rejection	5.221	1	0.0223 *	2.237	1	0.1347	3.367	1	0.0665 *	0.365	1	0.5456
Body contact	1.501	1	0.2205	0.114	1	0.7357	1.751	1	0.1857	0.410	1	0.5222
Social play	0.287	1	0.5921	0.007	1	0.9348	0.260	1	0.6104	1.049	1	0.3057
Grooming	0.006	1	0.9408	3.329	1	0.0681 †	0.367	1	0.5447	1.115	1	0.2910
Being groomed	0.021	1	0.8849	3.779	1	0.0519 †	0.225	1	0.6354	0.356	1	0.5508
Vocalization	0.011	1	0.9178	3.158	1	0.0755	0.803	1	0.3701	2.350	1	0.1253
Locomotion	5.946	1	0.0147 *	7.626	1	0.0058 **	1.483	1	0.2233	0.871	1	0.3507
Eating	1.897	1	0.1684	1.624	1	0.2025	0.008	1	0.9293	0.040	1	0.8414
Drink	0.417	1	0.5183	0.094	1	0.7591	1.390	1	0.2384	2.063	1	0.1509
Sleep	4.230	1	0.0397 *	2.081	1	0.1492	2.696	1	0.1006	3.809	1	0.0510 †
Yawn	0.254	1	0.6142	0.210	1	0.6469	0.432	1	0.5111	0.403	1	0.5256
Self scratch	0.557	1	0.4555	1.354	1	0.2445	0.110	1	0.7397	0.450	1	0.5024
Self grooming	0.508	1	0.4759	0.584	1	0.4449	0.629	1	0.4279	0.393	1	0.5308
Stereotypy	4.221	1	0.0399 *	1.937	1	0.1640	1.089	1	0.2966	2.711	1	0.0997 †

Supplementary Table 6 Results of statistical analysis in infant retrieving assay with amylin or AC187 infusion into the cMPOA (related to Fig. 5)

	amylin			AC187		
	t value	Df	p value	t value	Df	p value
Retrieving latency	-0.8206	5	0.4492	-0.87181	7	0.4122
Rejection rate	-2.8007	5	0.03796 *	2.2454	7	0.0596 †
Carrying rate	2.694	5	0.04309 *	-1.6825	7	0.1364

Supplementary Table 7 Results of statistical analysis in family observation assay with amylin or AC187 infusion into the cMPOA (related to Fig. 6)

	amylin			AC187		
	t value	Df	p value	t value	Df	p value
Carrying	-1.1942	5	0.2866	-2.1374	7	0.0699 †
Retrieval	-1.7461	5	0.1412	-1.3229	7	0.2275
Rejection	0.48795	5	0.6462	-0.79772	7	0.4512
Body contact	-0.36728	5	0.7284	0.35753	7	0.7312
Social play	NA	5	NA	-1.1365	7	0.2932
Grooming	0.75207	5	0.4859	-0.07198	7	0.9446
Being groomed	1	5	0.3632	-1.4547	7	0.1891
Vocalization	0.15931	5	0.8797	-1.1104	7	0.3035
Locomotion	0.70201	5	0.514	-0.11952	7	0.9082
Eating	0.18319	5	0.8618	0.74387	7	0.4812
Drink	0.79057	5	0.465	0.60698	7	0.563
Sleep	-0.22041	5	0.8343	-1	7	0.3506
Yawn	-2.0868	5	0.09127 †	NA	7	NA
Self scratch	-0.45091	5	0.6709	0.12309	7	0.9055
Self grooming	1	5	0.3632	1.3107	7	0.2313
Stereotypy	1.19	5	0.2875	0	7	1

Supplementary Table 8 Results of statistical analysis in family observation assay with atosiban infusion into the cMPOA (related to Fig. 7)

	t value	Df	p value	
Body contact	-3.5081	3	0.0393	*
Social play	0.3974	3	0.7177	
Grooming	1	3	0.3910	
Being groomed	1	3	0.3910	
Vocalization	NA	3	NA	
Locomotion	-0.0245	3	0.9820	
Eating	2.4495	3	0.0917	
Drinking	1	3	0.3910	
Sleep	1	3	0.3910	
Yawn	1	3	0.3910	
Self scratch	2.1768	3	0.1177	
Self grooming	0.3974	3	0.7177	
Stereotypy	1	3	0.3910	
Marking	-1	3	0.3910	
Object play	-1	3	0.3910	

Supplementary Table 9 Results of statistical analysis of infant-directed behaviors with atosiban infusion into the cMPOA (related to Supplementary Fig. 2)

	t value	Df	p value
Retrieval latency	-0.5182	2	0.6559
Rejection rate	-1.9597	2	0.1891
Carrying rate	0.5705	2	0.6259
Carrying (family)	-0.6547	2	0.5799
Retrieval (family)	-2	2	0.1835
Rejection (family)	1.5119	2	0.2697

Supplementary Fig. 1 Cannulated marmoset's brain sections showing the site of microinfusion with trypan blue (related to Fig. 1)

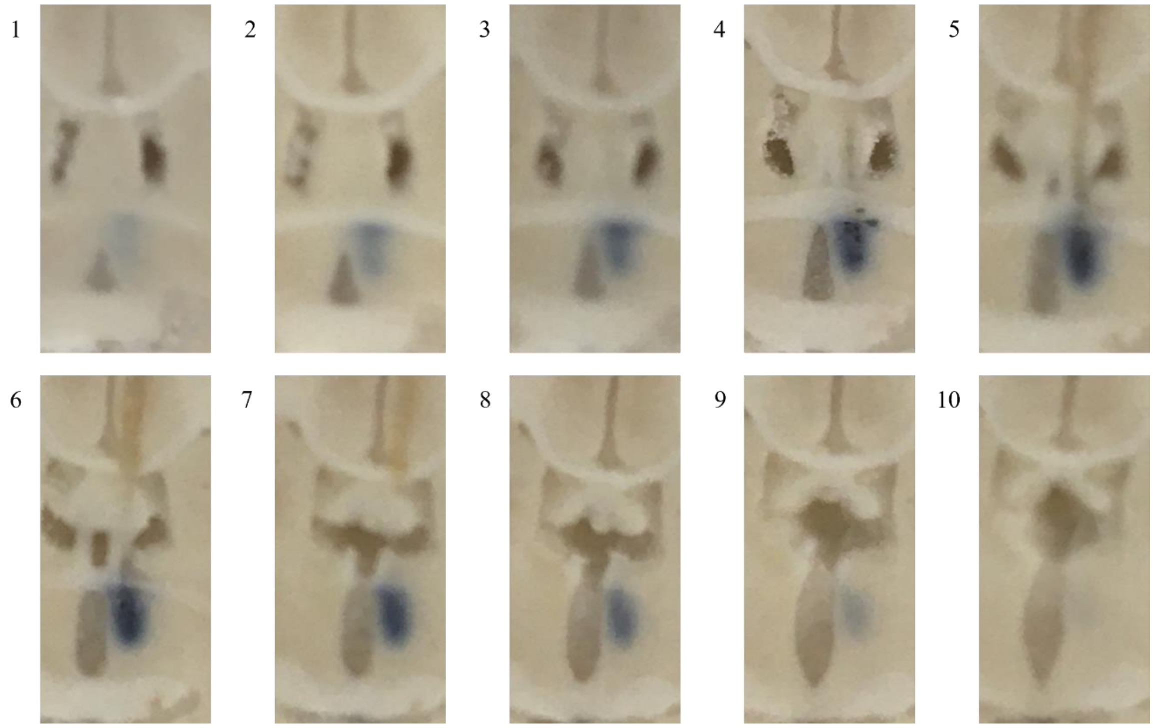

Trypan blue was injected before perfusion to detect the microinfusion site. Every 2nd section was photographed from 1 (anterior) to 10 (posterior).

Supplementary Fig. 2 The effects of atosiban infusion on infant-directed behaviors (related to Fig. 7)

<Infant retrieval assays>

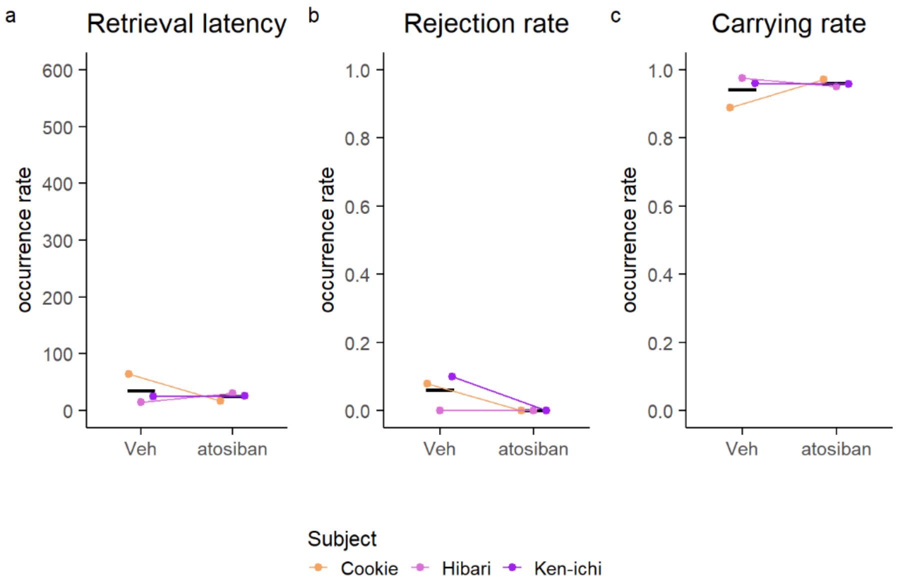

<Family observation>

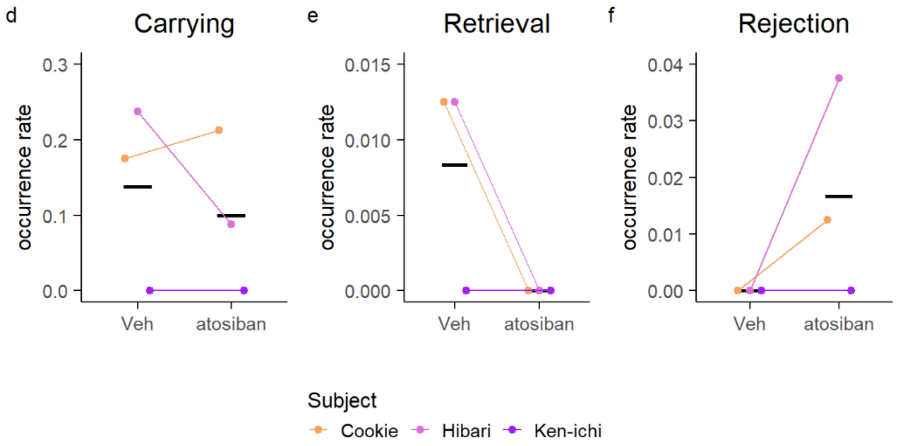

The infant-directed behaviors of subjects received atosiban infusion in the infant retrieval assays (a-c) and family observation (d-f). Each dot corresponds to the mean of each subject. The black horizontal lines show the mean values of each condition of all subjects. Statistical analysis was not performed because of the low sample numbers (N=3 each) due to the survival failure of Brian's younger siblings. Note that the Y axes of family observation parameters are variable, and both retrieval and rejection are rare.
