# Supplementary material for: Distinct roles of amylin and oxytocin signaling in intrafamilial social behaviors at the medial preoptic area of common marmosets
Source: Commun Biol. 2023 Dec 5;6:1231. doi: 10.1038/s42003-023-05593-5 (PMC10698028; doi:10.1038/s42003-023-05593-5)
Supplement: Supplementary file 3 — Description of Additional Supplementary Files [file 42003_2023_5593_MOESM3_ESM.pdf]

Description of Additional Supplementary Files

File name: Supplementary Data

Description: The source data behind the graphs in Figures 2, 3, 5, 6 and 7, and Supplementary Figure 2
